# Supplementary material for: Comparative therapeutic efficacy of remdesivir and combination lopinavir, ritonavir, and interferon beta against MERS-CoV
Source: Nat Commun. 2020 Jan 10;11:222. doi: 10.1038/s41467-019-13940-6 (PMC6954302; doi:10.1038/s41467-019-13940-6)
Supplement: Supplementary file 1 — Supplementary Information [file 41467_2019_13940_MOESM1_ESM.pdf]

**Supplementary Information**

# Comparative therapeutic efficacy of remdesivir and combination lopinavir, ritonavir, and interferon beta against MERS-CoV

Sheahan *et al.*

## Supplementary Figures

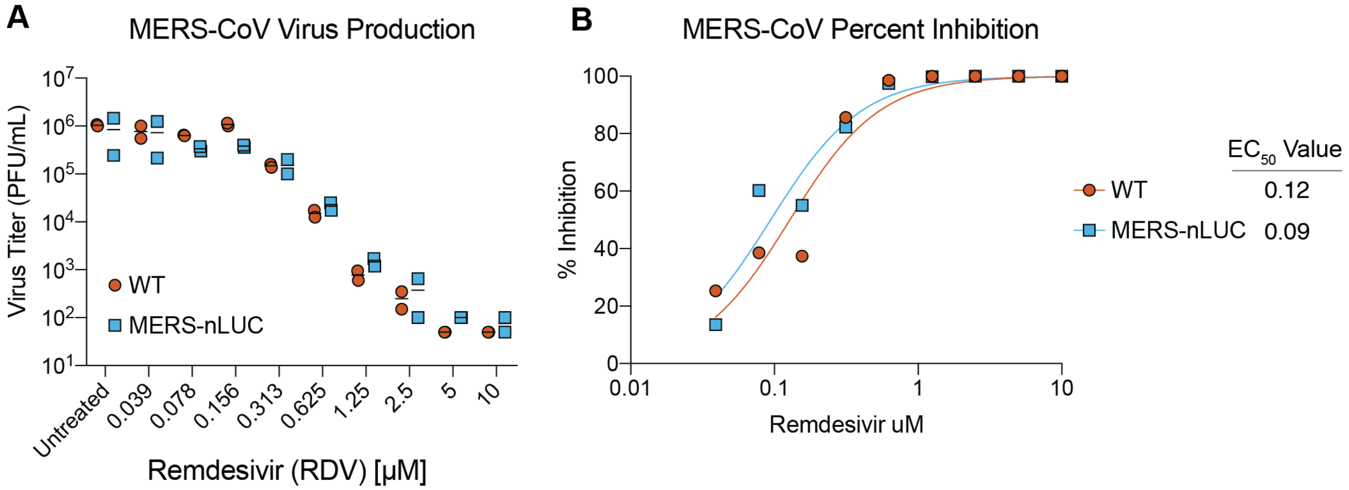


**Supplementary Figure 1. Antiviral assays with WT MERS-CoV EMC2012 and MERS-CoV expressing nLUC.** Calu3 2B4 cells were infected with WT EMC 2012 or MERS-nLUC at a multiplicity of infection (MOI) of 0.1 for 1 hour at 37˚C after which cultures were washed and a dose response of RDV diluted in two-fold steps in media (DMEM, 10% FBS, DMEM, 1x A/A) was added in duplicate. Cultures were then incubated at 37˚C in 5% CO2 for 24hr after which 100μl of media from each well was collected and assayed for virus production by plaque assay in Vero CCL81 cells. **(A)** MERS-CoV virus production across the RDV dose response. **(B)** $IC_{50}$ curves as defined in GraphPad Prism 7 (GraphPad).

## Supplementary Figures

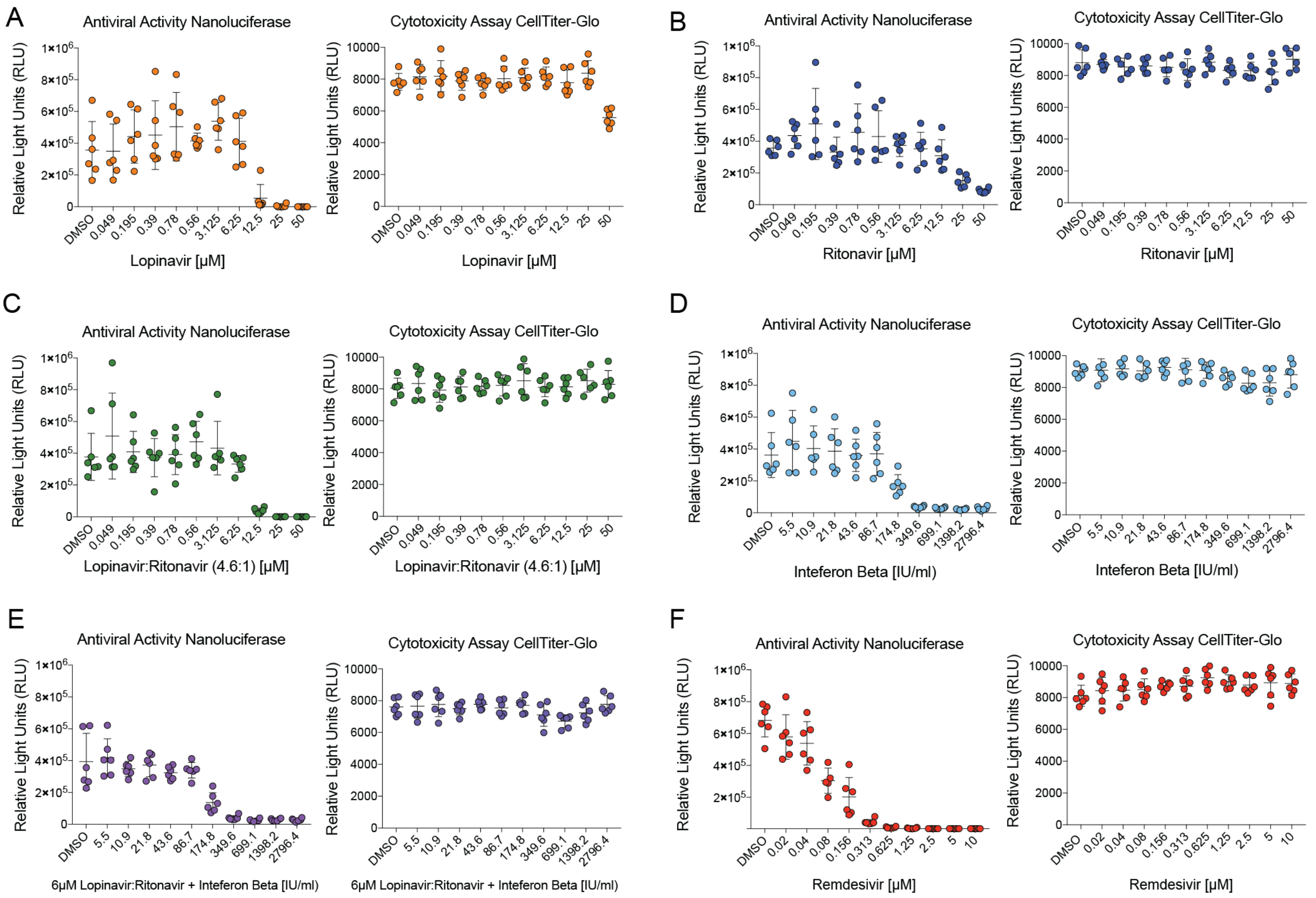


**Supplementary Figure 2. Example antiviral assay data in Calu-3 cells infected with MERS-CoV.** For each compound, we performed an antiviral assay and concurrently assessed cytotoxicity in non-infected sister plates as shown in **Figure 1**. Calu3 cells were infected in sextuplicate with MERS-nLUC and exposed to a dose response of compound and 48hr post infection virus replication was quantitated via nanoLuc assay. Sister plates were treated similarly with compound yet unexposed to virus and cell viability was measured via CellTiter-Glo assay 48hr after the initiation of treatment. **(A)** Lopinavir antiviral activity and cytotoxicity. **(B)** Ritonavir antiviral activity and cytotoxicity. **(C)** Combination lopinavir:ritonavir (4.6:1, molar ratio) antiviral activity and cytotoxicity. **(D)** Human interferon beta antiviral activity and cytotoxicity. **(E)** 6μM lopinavir:ritonavir (4.6:1, molar ratio) with a dose response of interferon beta. **(F)** Remdesivir antiviral activity and cytotoxicity. Each data point represents data from one well in a 96-well plate. Lines are at the mean and error bars represent the standard deviation. Similar data was achieved in three independent experiments.

## Supplementary Figures

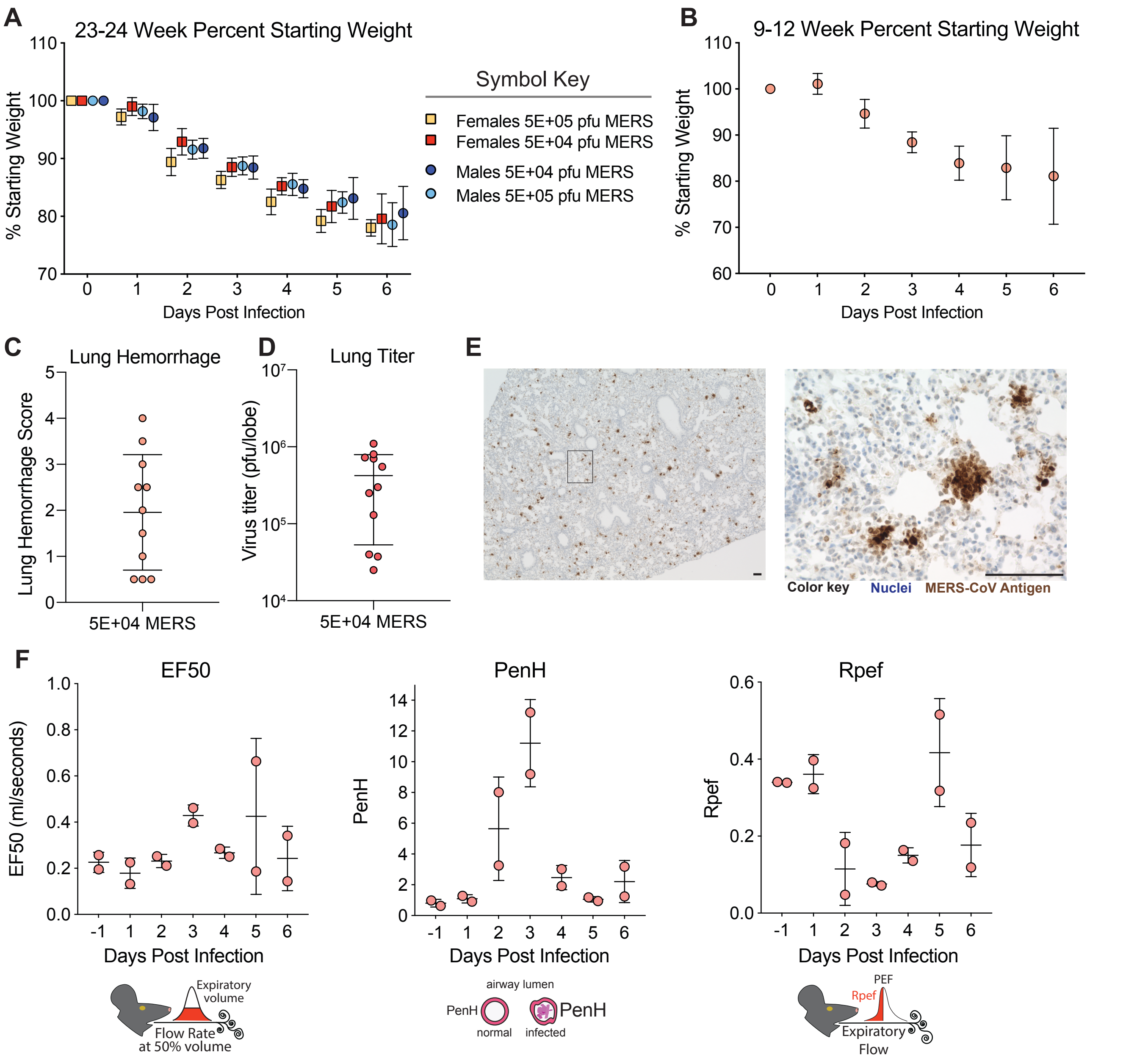


**Supplementary Figure 3. MERS-CoV pathogenesis and replication in Ces1c$^{-/-}$ *hDPP4*. (A)** Percent starting weight for equivalent numbers (N = 9-10/sex/group) of 23-24 week old male and female *Ces1c$^{-/-}$ hDPP4* mice infected with either 5E+04 or 5E+05 pfu MERS-CoV M35C4. **(B)** Percent starting weight for 9-12 week old female *Ces1c$^{-/-}$ hDPP4* infected with 5E+04 pfu MERS-CoV M35C4. For **A** and **B**, the symbol represents the mean and the error bars represent the standard deviation. (**C)** Lung hemorrhage score 6 dpi scored on scale of 0-4 where 0 is a normal healthy lung and 4 is a diffusely discolored lung. **(D)** MERS-CoV lung titer 6 dpi in mice as described in **(B)**. **(E)** Photomicrographs of example MERS-CoV antigen staining (brown) and hematoxylin stained nuclei (blue) in mouse lung tissue sections from 6 dpi at 40x magnification. The black bar is 100μM. **(F)** WBP was used to assess pulmonary function in mice daily. EF50 is the flow rate at 50% expired volume. PenH is a surrogate measure of airway resistance. Rpef is the fraction of expiration before peak expiratory flow is reached. Altered EF50, PenH and Rpef indicate bronchoconstriction or airway obstruction. For C, D and F, the symbols represent the data from a single animal, the line is at the mean and the bars represent the standard deviation. Asterisks indicate statistical differences by two-way ANOVA with Sidek's multiple comparison test.

# Supplementary Figures

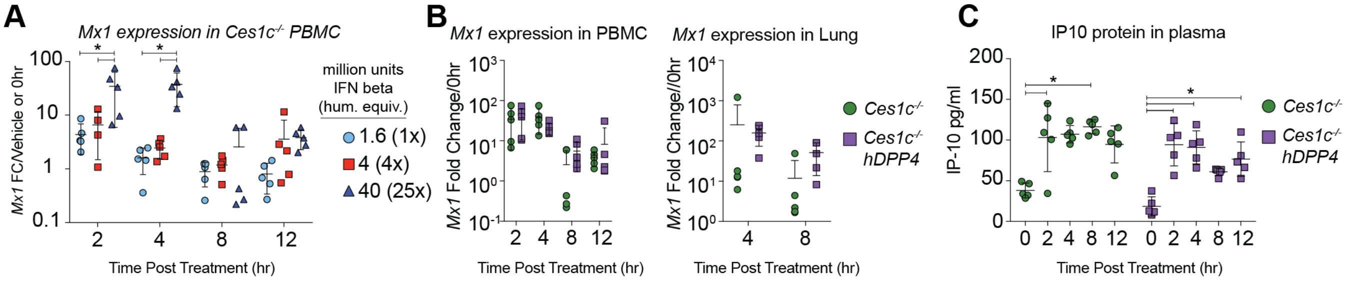


**Supplementary Figure 4. Peripheral administration of IFNb induces an ISG response in the lung.** 18-20 week old male and female *Ces1c*$^{-/-}$ mice were subcutaneously administered various doses of IFNb (R+D Systems) to better understand the biological effects of IFNb in the mouse strains in which we perform *in vivo* efficacy studies. **(A)** Interferon stimulated gene, *Mx1*, gene expression in PBMCs over time by qRT-PCR in *Ces1c*$^{-/-}$ mice administered 1X (1.6 MIU/kg), 2.5X and 25X human equivalent doses of IFNb in cohorts (N = 20-25/group) of Ces1c$^{-/-}$ mice. **(B)** Interferon stimulated gene, *Mx1*, gene expression in PBMCs over time by qRT-PCR in *Ces1c*$^{-/-}$ (N = 25) or *Ces1c*$^{-/-}$ *hDPP4* (N = 25) cohorts administered a 25X human equivalent dose of IFNb (i.e. 40 M IU/kg) (Left). Interferon stimulated gene, *Mx1*, gene expression in lung tissue of mice (Right). **(C)** IFN-gamma inducible protein 10 (IP10, CXCL10) in plasma via ELISA in mice from **(B)**. Each data point represents data from one mouse. Lines are at the mean and error bars represent the standard deviation. Asterisks indicate statistically significant differences according to two-way ANOVA with Sidek's multiple comparison test in **(A)** or one-way ANOVA with a Kruskall-Wallace multiple comparison test for **(C)**.

## Supplementary Figures

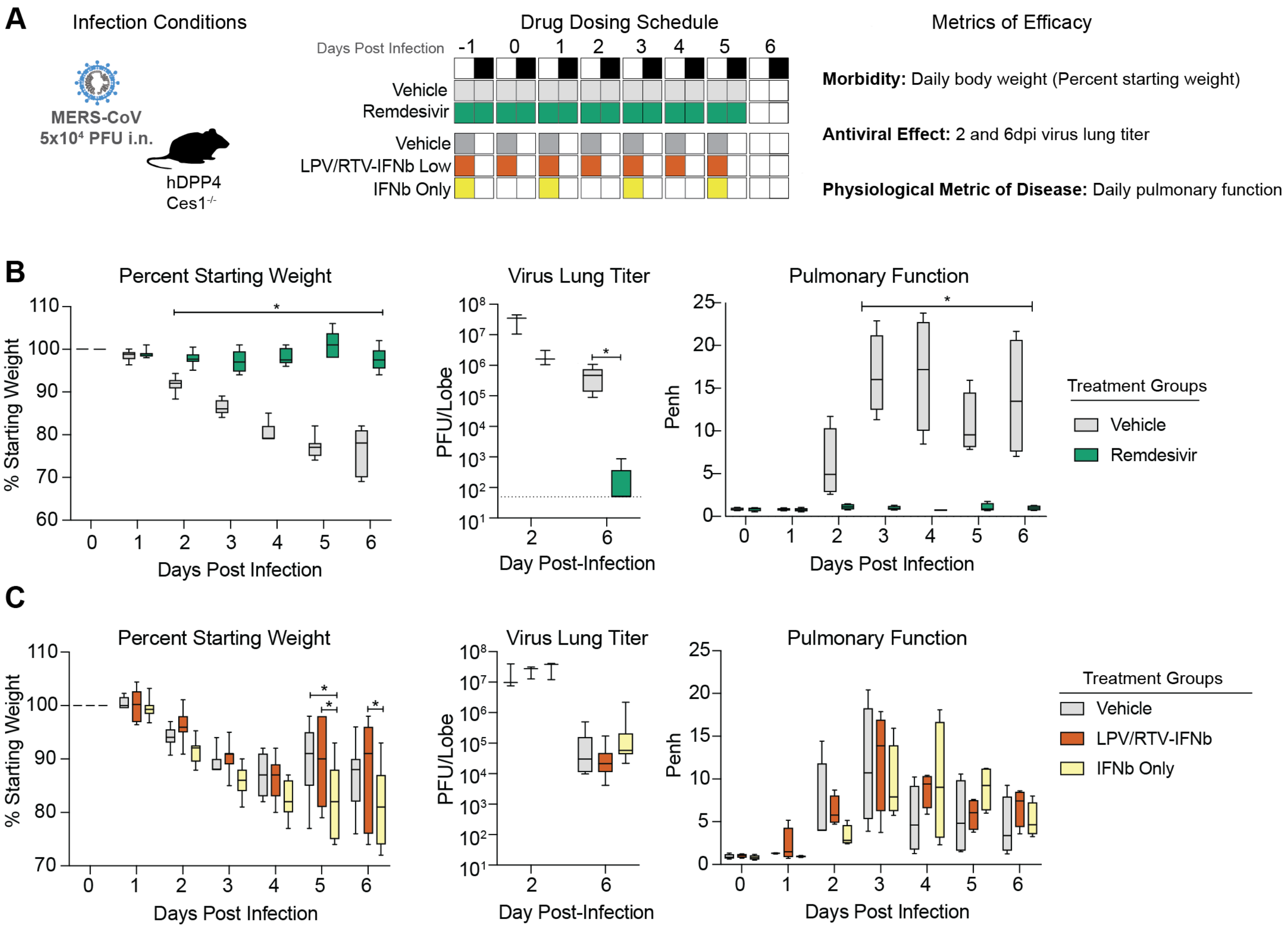


**Supplementary Figure 5. RDV provides superior prophylactic efficacy as compared to LPV/RTV+IFNb. (A)** Description of infection/drug dosing conditions and metrics of efficacy. **(B)** Percent starting weight (Left) of 11-13 week old female *Ces1c⁻/⁻ hDPP4* mice infected with 5E+04 pfu MERS M35C4 and treated BID with either vehicle (n = 9=10) or remdesivir (RDV, 25mg/kg, n = 9) subcutaneously beginning -1dpi. Asterisks indicate statistically significant differences ($P < 0.05$) as determined by two-way ANOVA and Tukey's multiple comparison test. (Middle) MERS-CoV lung titer on 2 (N = 3) and 6 dpi (all remaining animals). Asterisks indicate statistically significant differences ($P < 0.05$) as determined by two-way ANOVA and Sidek's multiple comparison test. (Right) WBP was used to assess pulmonary function in mice. PenH is a surrogate measure of airway resistance or bronchoconstriction. Asterisks indicate statistical differences by two-way ANOVA with Sidek's multiple comparison test. **(C)** Percent starting weight (Left), virus lung titer (Middle) and pulmonary function metric PenH (Right) of cohorts of mice similar in age and sex and infected similarly with MERS-CoV as in **B** but treated with vehicle (n = 10), LPV/RTV+ IFNb low (1x human equivalent) (n = 10), or IFNb low only (n = 10). Oral vehicle or lopinavir/ritonavir (160/40 mg/kg) were administered orally once daily beginning the -1dpi. IFNb treatment was initiated 24hr prior to infection and every other day thereafter. To control for dosing effects, vehicle treated mice received both oral vehicle and subcutaneous PBS to mirror IFNb injections. Similar statistical tests performed on **B** were performed on **C**. For **B** and **C**, the boxes encompass the $25^{th}$ to $75^{th}$ percentile, the line is at the median, while the whiskers represent the range.

## Supplementary Figures

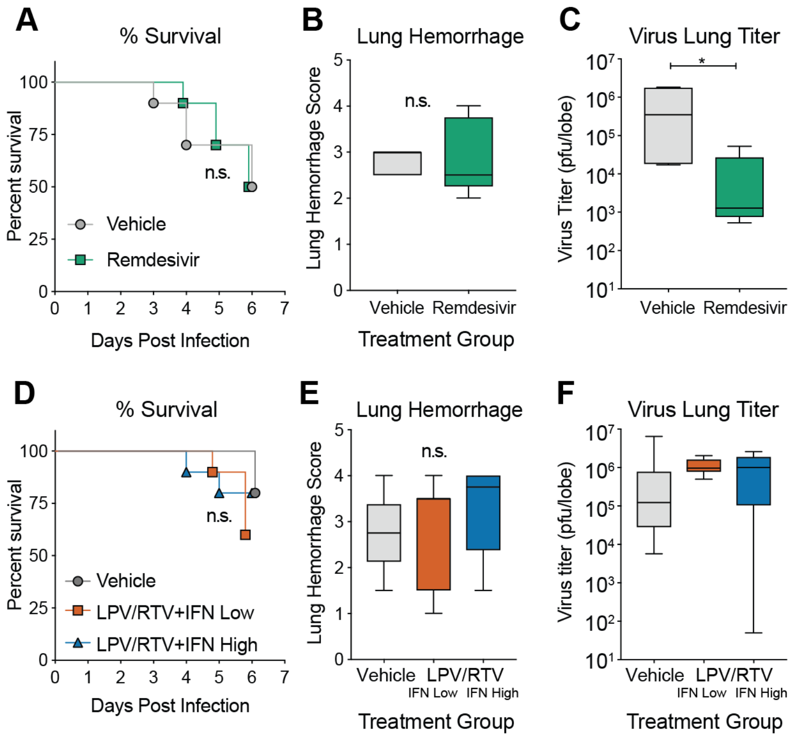


**Supplementary Figure 6. Therapeutic treatments do not improve survival with lethal MERS-CoV challenge.** Cohorts (n = 10) of 23-25 week old male and female *Ces1c*$^{-/-}$ *hDPP4* (5 female/5 male) mice were infected with 5E+05 pfu MERS-CoV M35C4 and started on antiviral treatment or vehicle 1dpi. **(A)** Percent survival of each cohort and survival analysis by Mantel-Cox test. Animals were treated subcutaneously BID with either vehicle or remdesivir as in Figure 5. **(B)** Lung hemorrhage score for animals in **A**. **(C)** Virus lung titer for animals in **A** 6dpi. Asterisk indicates statistical significance by Mann-Whitney P = 0.03. **(D)** Percent survival of each cohort and survival analysis by Mantel-Cox test. Animals were treated with either vehicle or LPV/RTV coupled with either low or high IFN beta as described in **Figure 5. (E)** Lung hemorrhage score for animals in **D. (F)** Virus lung titer for animals in **D** 6dpi.
